# PDE4-Mediated cAMP Signalling

**DOI:** 10.3390/jcdd5010008

**Published:** 2018-01-29

**Authors:** Bracy A. Fertig, George S. Baillie

**Affiliations:** University of Glasgow, Glasgow G12 8QQ, UK; b.fertig.1@research.gla.ac.uk

**Keywords:** phosphodiesterase 4, cardiac myocyte, vascular endothelium

## Abstract

cAMP is the archetypal and ubiquitous second messenger utilised for the fine control of many cardiovascular cell signalling systems. The ability of cAMP to elicit cell surface receptor-specific responses relies on its compartmentalisation by cAMP hydrolysing enzymes known as phosphodiesterases. One family of these enzymes, PDE4, is particularly important in the cardiovascular system, where it has been extensively studied and shown to orchestrate complex, localised signalling that underpins many crucial functions of the heart. In the cardiac myocyte, cAMP activates PKA, which phosphorylates a small subset of mostly sarcoplasmic substrate proteins that drive β-adrenergic enhancement of cardiac function. The phosphorylation of these substrates, many of which are involved in cardiac excitation-contraction coupling, has been shown to be tightly regulated by highly localised pools of individual PDE4 isoforms. The spatial and temporal regulation of cardiac signalling is made possible by the formation of macromolecular “signalosomes”, which often include a cAMP effector, such as PKA, its substrate, PDE4 and an anchoring protein such as an AKAP. Studies described in the present review highlight the importance of this relationship for individual cardiac PKA substrates and we provide an overview of how this signalling paradigm is coordinated to promote efficient adrenergic enhancement of cardiac function. The role of PDE4 also extends to the vascular endothelium, where it regulates vascular permeability and barrier function. In this distinct location, PDE4 interacts with adherens junctions to regulate their stability. These highly specific, non-redundant roles for PDE4 isoforms have far reaching therapeutic potential. PDE inhibitors in the clinic have been plagued with problems due to the active site-directed nature of the compounds which concomitantly attenuate PDE activity in all highly localised “signalosomes”.

## 1. Introduction

Cyclic 3′,5′-adenosine monophosphate (cAMP) was the first second messenger molecule to be discovered, and has been researched tirelessly in the context of numerous physiological systems. Much of the current understanding of cAMP signalling, however, has come from studying its function in the cardiovascular system, where it has major roles in the heart and vessels [1]. In the heart, cAMP influences a multitude of processes from contractility and hypertrophy of myocytes to apoptosis and cell survival [2]. In the vasculature, effects on smooth muscle cell contraction and relaxation, as well as endothelial cell permeability has been attributed to cAMP signalling processes [3]. Additionally, cAMP can modify cell proliferation, migration, differentiation, and response to stress [4,5,6,7]. cAMP produces these vast cellular effects by activating four types of effector proteins: protein kinase A (PKA), exchange protein directly activated by cAMP (EPAC), cyclic nucleotide activated ion channels (CNGC), and popeye domain containing proteins (POPDC). The present review will focus on the effects of PKA and EPAC. PKA functions to phosphorylate substrate proteins, while EPAC activates the RAS superfamily of enzymes [8]. The present review will focus on the ways in which phosphodiesterase 4 (PDE4) enzymes modify cAMP’s ability to produce these varied physiological effects within the cardiovascular system.

## 2. cAMP Signalling and Compartmentalisation 

The small, highly diffusible molecule, cAMP can be produced by both membrane-bound adenylyl cyclase (mAC) and soluble adenylyl cyclase (sAC) in response to various stimuli, such as the activation of various G_s_-coupled receptors, which activates mAC [9]. Its properties suggest that it would quickly diffuse throughout the cell, simultaneously activating all effector proteins almost instantly. In striking contrast, it was shown by Larry Brunton and colleagues in the early 1980s that cAMP can cause multiple discrete receptor specific responses in the same cells [10,11]. To explain this phenomenon, it was quickly postulated, and eventually proven, that compartmentalisation of cAMP signalling underpins receptor specific responses by restricting the number and identity of PKA substrates that get phosphorylated in response to each specific receptor ligation [12,13]. Such fine control of one ubiquitous second messenger that acts to activate only discrete pools of PKA is made possible by the subcellular localisation of proteins that degrade cAMP. This function is attributed to a super-family of enzymes called phosphodiesterases (PDEs). PDEs are the only known route to the hydrolysis of cAMP and this function empowers these enzymes to act as “sinks”, reducing cAMP concentration in localised areas preventing the inappropriate phosphorylation of PKA substrates under basal conditions. Following receptor activation, however, this situation can be altered to allow cAMP concentrations to exceed the activation threshold of PKA enzymes tethered to discretely positioned “signalosomes”. This situation occurs only when the cAMP concentration in the vicinity of the relevant “signalosomes” is high enough to swamp the PDE component, promoting downstream physiological effects [14]. The integration of PDE4 isoforms into specific “signalosomes” within the heart and the function of these protein complexes will be the subject of this review.

## 3. PDEs and PDE4-Ology

**Figure 1:**
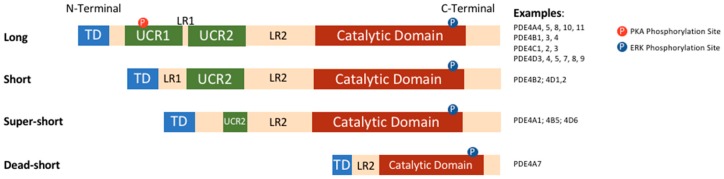
Diversity of domain organisation of PDE4 isoforms TD, transduction domain; LR1/2, linker region 1/2; UCR1/2, upstream conserved region 1/2.

PDEs are a large super-family of enzymes, which are the products of 11 different gene families, grouped according to their structure, function, and affinity for cAMP and cGMP. Structurally, all PDEs have conserved carboxy-terminal catalytic cores while their amino-terminal regions differ among families, subfamilies, and specific isoforms. The N-terminal regions have a number of functional roles. These include the targeting to specific subcellular locations and to signalosomes, and the modulation of responses to signals from regulatory molecules or post-translational modifications [1,15]. cAMP-specific PDE4s make up the largest family with over 20 isoforms encoded by four genes (A, B, C, and D). Each isoform has a unique N-terminal region, made up of an N-terminal targeting domain (TD). Additionally, the N-terminal region contains upstream conserved regions 1 and 2 (UCR1 and UCR2), which are linked to each other and to the catalytic domain by linker region 1 and 2 (LR1 and LR2) respectively [16]. Based on the presence and size of UCR1 and UCR2, PDE4 isoforms can be categorised into long, short, super-short, and dead-short isoforms (Figure 1); long isoforms have both UCR1 and 2, short isoforms have only UCR2, super-short have a truncated UCR2, and dead-short isoforms lack both UCR domains and have a truncated catalytic domain [17]. 

UCR1 and 2 have a number of roles, including the regulation of functional outcomes following PDE4 phosphorylation by protein kinase A (PKA) or extracellular signal-regulated kinase (ERK). The presence or absence of these domains means that phosphorylation can have different, and even opposite, functional effects in different isoforms [18,19,20].

The catalytic domain of PDE4 has been extensively studied, and X-ray crystal structures have demonstrated the deep hydrophobic pocket made of numerous helices which makes up the active site for the hydrolysis of cAMP [15,21]. The structure of this domain has been integral for the design of family-specific inhibitors, which have been widely used both clinically and experimentally [22,23]. 

As previously mentioned, the subcellular localisation of PDEs is integral to their function in shaping cAMP gradients and responses. This localisation is often directed by the N-terminal targeting domains, which are highly varied among PDE4 isoforms [24,25], though targeting can also be driven by other regions including the newly identified multi-functional docking domain positioned at the C-terminal end of the catalytic unit [26]. Unique subsets of PDE4 specific interactions allow single isoforms to have multiple non-redundant roles in different tissues, cells and even different micro-domains within the same cell [27]. The disruption of this location dependent function of small pools of highly active PDE4s has been considered as a novel way to circumvent side-effects observed following the systemic inhibition of global PDE4 activity using site-directed PDE4 inhibitors [28].

## 4. Technological Approaches to Defining Roles for PDE4 Isoforms

Isoform specific roles of PDEs have been identified within the realm of the cardiovascular system and historically, as new technologies have been invented, these have been used to identify specific interactions and functions of PDE4. Often, the first and easiest approach utilised is pharmacological, and employs family-specific inhibitors. However, this strategy gives little information on individual isoforms and can often engender “bulk” cAMP effects due to simultaneous inhibition of all PDE4 enzymes in all locations. Other more targeted approaches include siRNA knockdown and overexpression of dominant-negative PDEs. Both of these allow diminution of the influence of one isoform by either genetic silencing or displacement of endogenous, functional PDEs [29,30,31]. Often these approaches are sufficient to pinpoint specific cellular functions for single isoforms; however, if one isoform has multiple roles in the same cell, an incomplete understanding of the complexity of PDE4 action can be lost.

The identification of specific protein-protein interactions within PDE signalosomes has been integral to understanding PDE4 function. Peptide array technology has proved to be an accurate way to screen for such interactions and to predict the regions and specific amino acids which are essential for holding signalling complexes together [32,33,34]. Another advantage of this technique is that it allows the informed design of cell-permeable disruptor peptides, which can interrupt these signalosome interactions. Disruptor peptides provide by far the most specific form of inhibition as only a specific localised pool of the PDE4 isoform of choice is displaced, leaving any other cellular effects of that isoform untouched [28,35].

The development of fluorescent probes which can monitor cAMP dynamics in real time have led to vast advancements in the field. Briefly, these probes contain two fluorescent moieties, a donor and an acceptor, and a cAMP binding domain. When cAMP binds, a conformational change is induced, altering the fluorescent energy transfer between the donor and acceptor, known as fluorescence resonance energy transfer (FRET). The detection of the two emissions allows real time changes in cAMP concentration to be measured [36].

Many of the studies described in the present report employ a combination of these techniques to create robust evidence for the specific roles of PDE4 isoforms in the cardiovascular system.

## 5. PDE4 in the Heart

Much of the understanding of cAMP compartmentalisation and the role of PDEs comes from extensive studies of the signalling pathway in cardiac myocytes. Generally speaking, four PDE families have been identified as having notable roles in cAMP signalling in the heart: PDE1, PDE2, PDE3, and PDE4 [37]. Despite the identification of localised expression of PDE4 in cardiac myocytes, inhibition of this family was shown to have very limited effects on cardiovascular parameters, such as basal blood pressure, heart rate, and contractility. This can be explained by the fact that PDE4 is a major player not at basal cAMP levels, but during β-adrenergic stimulation, when intracellular [cAMP] is raised. Additionally, it has been shown that redundancy of the PDE network can lead to compensatory effects, in which other families with the same hydrolytic activity attenuate the effects of PDE4 loss of function [38]. 

In the cardiac myocyte, sympathetic activation leads to β-adrenergic signalling, a major part of the fight or flight response. Briefly, when the receptor is activated, its associated G_s_-protein causes the activation of AC, which catalyses the production of cAMP from ATP. cAMP in turn activates various effector proteins; of major importance for this signalling paradigm is PKA, which phosphorylates a number of substrates important for cardiac excitation-contraction coupling, and EPAC, which is of particular importance in the vasculature. These phosphorylation effects lead to positive inotropic and lusitropic effects, increasing the function of the heart for increased circulatory demands [39].

Interestingly, the phosphorylation of these substrate proteins by PKA is tightly regulated by PDE4 enzymes, which associate directly within signalosomes, modifying the signalling in tight compartments. In this way, it is not global signalling which is affected by PDE4, but local, compartmentalised signalling. This concept, developed in the 1980s by Hayes et al. using biochemical means was proven unequivocally using advanced imaging techniques [12]. Optical probes that acted as cAMP FRET reporters, allowed visualisation of the formation of cAMP gradients following submaximal β-adrenergic stimulation. When used in combination with PDE inhibitors, it became apparent that the loss of cAMP hydrolysis within cellular microdomains abrogated the spatial and temporal control of cAMP dynamics that is required to channel receptor-specific physiological outcomes [13,40].

PDE4 and cardiac disease have a complicated relationship [41]. On one hand, PDE inhibition has been shown to enhance cAMP signalling, resulting in increased cardiomyocyte function. In contrast, chronic inhibition of PDEs results in increased mortality, often due to cardiac side effects [41,42]. Finally, PDE4 activity is decreased in heart failure, potentially contributing to disease progression due to faulty regulation of the sympathetic induction of phosphorylation of cardiac excitation-contraction coupling proteins [43,44].

**Figure 2:**
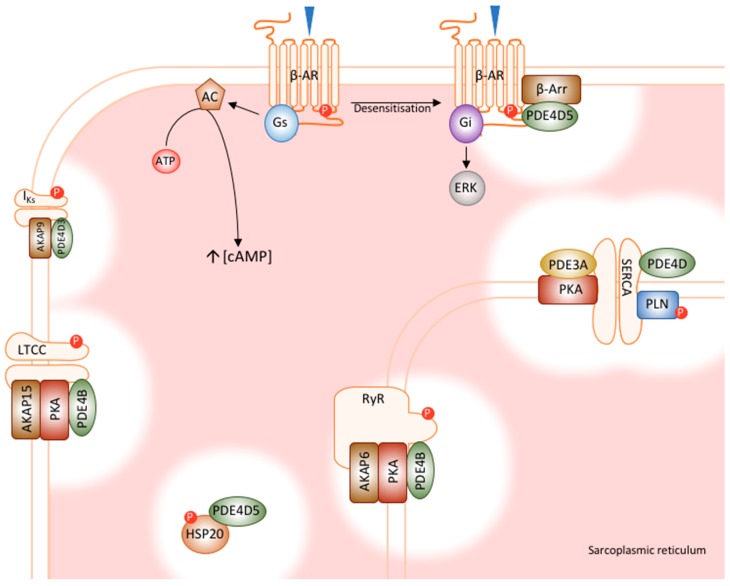
Cardiac PDE signalosomes. PDE4 family members have been shown to integrate into macromolecular complexes with numerous cardiac proteins, many of which are involved in excitation-contraction coupling and calcium handling. PDE4D5 interacts with β-arrestin, regulating receptor desensitisation. PDE4B regulates both the LTCC and RyR, which controls the process of calcium induced calcium release. PDE4D interacts with SERCA, regulating the reuptake of calcium into the SR. PDE4D3 interacts with IKs, modulating basal channel function. Finally, PKE4D5 regulates the cardioprotective effects of HSP20. AC, adenylyl cyclase; AKAP, A-kinase anchoring protein; ATP, adenosine triphosphate; β-AR, β-adrenoceptor; β-Arr, β-arrestin; ERK, extracellular signal regulated kinases; G_i_; inhibitory G-protein; G_s_, stimulatory G-protein; HSP20, heat shock protein 20; I_KS_, cardiac I_KS_ potassium channel; LTCC, L-type calcium channel; P, phosphorylation; PKA, protein kinase A PLN, phosphopamban, RyR, ryanodine receptor SERCA, sarcoplasmic reticulum calcium ATPase [35,43,45,46,47,48,49].

The role of PDE4 in the cardiac myocyte cannot be described in full without delving into the discrete roles of specific isoforms within their discrete subcellular compartments. The next sections of this review will focus on these unique functions. A schematic summary of cardiac PDE signalosomes is shown in Figure 2.

### 5.1. PDE4D5’s Role in β-Adrenoceptor Desensitisation

One of the best understood functions of PDE4 in the cardiovascular system is the role of PDE4D5 in the regulation of β_2_-adrenergic signalling in the cardiac myocyte. When the β_2_-adrenoceptor is stimulated, its coupled G_s_ protein stimulates AC, which produces cAMP to activate localised PKA. Desensitisation of adrenergic signalling occurs when a negative-feedback loop is created when the receptor itself is phosphorylated by PKA. This phosphorylation switches the receptor’s signalling to an inhibitory G protein, G_i_, which inhibits AC as well as activating other pathways, such as ERK1/2 [50]. Additionally, the phosphorylation of the receptor by G-protein coupled receptor kinases (GRKs) causes the recruitment of β-arrestin, which further desensitises the receptor by sterically hindering access to the G-protein [51]. However, when it was observed that the recruitment of β-arrestin also increased local cAMP degradation, it became clear that PDE translocation was a crucial function of β-arrestin [52]. In 2003, Baillie and colleagues used pharmacological inhibition and overexpression of dominant negative mutants to show that PDE4D5 is recruited to the β_2_-adrenoceptor by β-arrestin. In neonatal rat ventricular myocytes (NRVM), both rolipram (a PDE4 inhibitor) and dominant negative PDE4D5 increased the PKA phosphorylation of the receptor, and the activation of ERK1/2, which is consistent with phenotypic switching of the receptor [53]. In a similar study, siRNA was used to knockdown pan-PDE4D, which caused similar effects, namely, increased PKA phosphorylation of the receptor and ERK activation. The same effects were achieved by the specific knockdown of PDE4D5 and this was the first time a function had been ascribed to an individual PDE4 isoform [29].

Interestingly, PDE4D5 is not the only isoform which has been shown to interact with β-arrestin. In fact, all PDE4 isoforms have this ability due to the presence of the binding site in the conserved catalytic region [52]. The presence of an additional β-arrestin binding site on PDE4D5’s unique N-terminal binding domain could confer the specificity of its functioning as it is the “chosen-one” for β-arrestin translocation [46]. Additionally, other pools of PDE4 which are expressed in the cell could be preferentially sequestered in a different cellular location with different binding partners.

The direct interaction of the PDE with β-arrestin, however, is not sufficient to explain these observations. The localisation of a pool of PKA to the β_2_-adrenergic receptor has been shown to depend on the scaffolding protein, AKAP79 [29]. This allows the tethered PDE to regulate the activity of this discrete pool of PKA. This determines the phosphorylation status and, thereby, the phenotypic switching of the receptor.

### 5.2. PDE4D5 Regulates Cardioprotection by HSP20

Another example in which PDE4 has been shown to regulate the phosphorylation of a crucial cardiac signalling protein is in the case of the small heat shock protein 20 (HSP20, HSPB6). HSP20 is part of a diverse, ubiquitously expressed family of small chaperone proteins, whose expression patterns are regulated by cellular stressors [54]. HSP20 has been shown to have a protective role in cardiac myocytes, but only in its phosphorylated state [55,56,57]. In fact, when HSP20 is phosphorylated by PKA at Ser16, it protects the cell by switching off harmful signalling (NF-kB) and switching on protective signalling (Akt/PKB), inhibiting necrosis and apoptosis, and stabilising the cell’s cytoskeleton [58,59,60,61]. These protective mechanisms have been studied in the context of a variety of cardiac indications, including ischaemia/reperfusion, chronic β-adrenergic stimulation, and heart failure [61,62,63]. The importance of phosphorylation has been shown in a variety of mutant studies, in which a non-phosphorylated mutant (S16A) lost its protective effects in I/R injury, and constitutively phosphorylated mutant (S16D) effectively protected cells from apoptosis [55,56].

The importance of PKA phosphorylation for HSP20’s cardioprotective abilities led researchers to investigate the role of PDEs in this signalling pathway [35]. Initial experiments showed that pharmacological inhibition of PDE4 augmented the isoprenaline-induced increase in phosphorylated HSP20, confirming the functional role of PDE4. Real time monitoring of cAMP levels using FRET technology gave more insight into PDE4’s role in this pathway. Comparing the response of a cAMP FRET probe tethered to HSP20 with an untethered, cytosolic probe showed that PDE4 inhibition caused a greater increase in cAMP in the vicinity of HSP20. This result highlights once again that the location of small active pools of PDE4 is key to the enzyme’s control of specific events in cardiac myocytes.

The next step was to determine whether there was a direct interaction. Co-immunoprecipitation studies were used in conjunction with peptide array experiments to show that HSP20 directly interacts with the conserved catalytic region of PDE4D isoforms. The identification of the amino acids important for the docking of HSP20 to PDE4D allowed the design and manufacturing of a cell permeable peptide (peptide 906) which dismantles the interaction. Treatment of NRVM with this peptide not only resulted in an increased phosphorylation of HSP20, but also an attenuation of hypertrophic response to sustained β-adrenergic stimulation [64]. The peptide was also subsequently used in a rodent model of heart disease with great effect [65].

### 5.3. PDE4D3’s Regulation of RyR Phosphorylation

In the clinic, the use of PDE inhibitors has been plagued with cardiac problems, including increased susceptibility to arrhythmia and heart failure. The underlying mechanisms were unknown until Lehnart and colleagues performed an elegant study using PDE4D deficient mice [43]. It was observed that these animals developed a dilated cardiomyopathy which had many characteristics consistent with human chronic heart failure as well as exercise induced ventricular arrhythmias (VT). Upon observation at the cellular level, it was shown that despite normal global cAMP signalling in the PDE4D^‒/‒^ mice, there was a marked increase in localised cAMP at the cardiomyocyte Z line. This observation coupled with the evidence of previous studies showing that the ryanodine receptor (RyR) is hyperphosphorylated by PKA at Ser2808 in heart failure led the researchers to study the role of PDE4’s regulation of RyR phosphorylation in their model [66,67,68,69]. Indeed, the PDE4D^‒/‒^ mice showed both hyperphosphorylation of the RyR as well as diminished levels of calstabin-2 (FKBP12.6), which functions to stabilise RyR and reduce calcium leak from the sarcoplasmic reticulum [70,71]. These changes led to multiple phenotypic observations. In terms of RyR function, the channels had a “leaky” phenotype, similar to that which has been shown in heart failure and exercise induced sudden cardiac death [66,70]. 

The individual isoform of PDE which associates with RyR to regulate these effects was identified as PDE4D3. This is consistent with PDE4D3 being one of the three PDE4D isoforms expressed in the heart; however, studies completed in rat hearts should be extrapolated with caution due to potential differences in expression patterns of human hearts [72]. The elimination of PDE4D3 in complex with RyR was shown to be highly relevant physiologically as PDE4D3 levels in the complex are also reduced in human heart failure samples. This provided an interesting explanation for the hyperphosphorylation of RyR by PKA despite globally decreased cAMP signalling in failing human cardiac myocytes, as had been previously observed [73]. 

As the final piece of the puzzle, the group sought to confirm the role of hyperphosphorylated RyR as a causative factor for the observed phenotypes. The PDE4D^‒/‒^ mice were crossed with mice harbouring a mutation which did not allow PKA phosphorylation of the RyR (S2808A). These mice were protected against both exercise induced sudden cardiac death and myocardial infarction (MI) induced sudden cardiac death, confirming the role of the PDE4D3, PKA, RyR signalosome in the observed phenotypes. 

The similarities of the phenotypes observed in PDE4D^‒/‒^ mice with human cardiac disease provide a compelling argument for the role of aberrant signalling relating to maladjustment of the PDE complement within cardiac “signalosomes” in cardiomyopathy. Additionally, the identification of PDE4D3 in complex with the RyR pinpointed another unique function for an individual PDE4 isoform.

### 5.4. PDE4D3 Regulates Basal I_Ks_ Activity

The electrical activity of cardiac myocytes is another function where PDE4 has an active input. The slowly activating potassium channel (IKs) is a major repolarising current in the cardiac action potential. IKs exists as a macromolecular complex which comprises KCNQ1 α-subunits and KCNE1 β-subunits which interact with a variety of proteins that regulate the channel’s function [74,75]. These include AC9, AKAP-9, PKA, and protein phosphatase-1 [76,77]. As with many other cAMP signalling models in the cardiac myocyte, it was clear that a PDE may be involved in this signalosome, thereby regulating PKA phosphorylation of the α-subunits. In a primarily biochemical study, immunoprecipitation techniques identified that PDE4D3 is spatially confined within this macromolecular complex via a direct interaction with AKAP-9 [49]. Functional experiments in ventricular myocytes using pharmacological inhibitors showed that the PDE4 was able to fine tune the functioning of the channel at basal conditions, probably due to a basally active AC in the vicinity.

### 5.5. PDE4B Has a Dominant Role in LTCC Regulation

The L-type calcium channel (LTCC) is yet another cardiac excitation-contraction coupling protein whose function is modified by a PDE4 isoform. This protein functions to allow calcium influx through the cardiac myocyte membrane, which triggers the release of calcium from intracellular stores by the RyR, in a process known as calcium-induced calcium release (CICR) [39]. Under conditions of β-adrenergic stimulation, PKA phosphorylates the pore forming subunits of the LTCC, which increases channel activity [78,79]. In combination with RyR phosphorylation, CICR is increased, leading to a positive inotropic effect [39]. 

Although both PDE4B and PDE4D were both shown to tether to the LTCC signalosome, a study using subfamily specific knockout mice showed that PDE4B exhibits the dominant functional role in the regulation of LTCC phosphorylation and the protection against arrhythmia [47]. Firstly, experiments in wild type mice showed that PDE4 inhibition had no effect on basal calcium current through the LTCC (I_Ca,L_) but markedly increased I_Ca,L_ under conditions of β-adrenergic stimulation. Consistently, in both PDE4B^‒/‒^ and PDE4D^‒/‒^ mice, I_Ca,L_ was increased, leading to increased calcium transients and increased contractility. The disease relevance of this research was shown using in vivo pacing in the knockout animals, which resulted in ventricular tachycardia (VT) in PDE4B^‒/‒^ mice but not in PDE4D^‒/‒^ mice. This study displayed the dominant functional role of PDE4B in the regulation of phosphorylation of LTCC, which consequently regulates the entry of calcium into the cell and the initiation of CICR.

The role of PDE4B in the protection against arrhythmia is consistent with the observation of decreased PDE4B activity in cardiac hypertrophy [44]. The combination of these two findings could represent at least a partial explanation of the incidence of arrhythmia and sudden cardiac death associated with heart failure.

### 5.6. PDE4D and PDE3A Regulate PLN Phosphorylation and SERCA Function

The regulation of sarcoplasmic reticular calcium ATPase (SERCA) function relies on both PDE3 and PDE4 isoforms, unlike in other cardiac PDE signalosomes. 

SERCA is a calcium channel on the sarcoplasmic reticular membrane which functions to sequester calcium in its intracellular store. Phospholamban (PLN) inhibits its activity, tightly regulating channel function. PLN can be phosphorylated by PKA, which reduces its inhibitory influence, leading to increased SERCA activity and enhanced sarcoplasmic reticulum (SR) calcium load and contractility [80].

Both PDE4D and PDE3A have been shown to integrate into a signalosome containing SERCA, PLN, PKA and other structural and regulatory proteins, thereby regulating PLN phosphorylation and exhibiting a regulation of basal contractile function [48,81]. In separate studies, members of both PDE subfamilies were detected in SERCA immunoprecipitates, showing that these PDEs integrate into the SERCA/PLN signalosome. Knockout of PDE3A in rodents resulted in increased phosphorylation of PLN associated with increased SERCA activity, SR calcium load, and increased contractility, suggesting a major role for this isoform in the SERCA microdomain. However, similar studies in PDE4D knockout rodents produced strikingly similar results, from increased PLN phosphorylation to increased contractility. Taken together, it is apparent that both PDE3A and PDE4D are important modulators of cAMP signalling at the SERCA microdomain as both seem to have comparable effects. Further studies will be necessary to dissect any unique, non-compensated roles of these PDE isoforms on SERCA function.

As evidenced by the examples above, highly localised pools of PDEs and in particular PDE4 have influence on many steps of the cardiac excitation-contraction coupling process. Although the identity of the PDE4 isoforms differ in the distinct locations, the underlying concept of dormant signalosomes being activated following the inhibition, displacement, silencing or overwhelming of a spatially restricted PDE4 cohort prevails. Clearly, the initial concept pioneered by Hayes et al. and subsequently validated using optical cAMP probes has also been upheld in diseases that occur as a result of the malformation of highly tuned cAMP gradients underpinned by loss of localised PDE4 machinery [12].

## 6. Vascular cAMP Signalling and PDEs

Although cardiac myocytes have taken centre stage in terms of PDE4 research, this group of enzymes also has major roles in the wider cardiovascular system, regulating many characteristics of the vasculature. cAMP signalling is particularly important in the signalling systems of vascular endothelial cells (VECs) where increases in cAMP concentration results in reduced vascular permeability. The literature is clear that PDEs are integral to the fine control of such processes [82]. In conditions of low cGMP, PDE4 is the major regulator of PKA and EPAC activation, both of which influence permeability. In addition to PDE4, PDE2 and PDE3 activity also impinge on VEC permeability, but their influence is changed by increases in cGMP, which activates PDE2 and inhibits PDE3, allowing fine tuning of regulation [82,83]. As in cardiac myocytes, localised signalosomes have been identified as crucial in the maintenance of signalling specificity. The components of compartmentalised signalling complexes in this location include various AC subtypes, PDE2, PDE3, and PDE4 isoforms, and AKAPs. An important study by Maurice and colleagues identified the unique, non-compensated role of PDE4D in the regulation of vascular permeability, confirming the notion of compartmentalised signalling in VECs [3,83].

### PDE4D’s Role in the Regulation of Vascular Permeability

**Figure 3:**
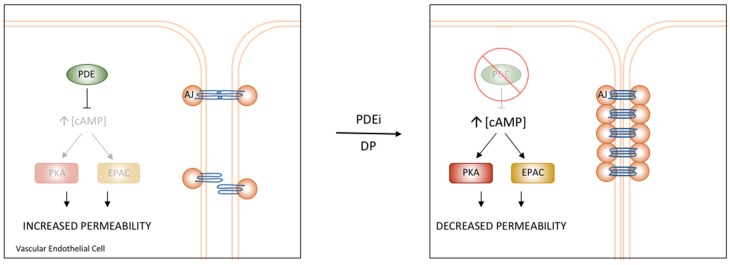
PDE regulation of Vascular Endothelial Permeability. PDE4D has been shown to regulate the permeability of the vascular endothelium through interactions with components of AJs. Treatment with PDE inhibitors or a specific disruptor of the PDE4D/EPAC1 interaction results in increased cAMP and stabilization of AJs, ultimately causing reduced permeability and increased barrier function [3].

VEC permeability is largely mediated through adherens junctions (AJ), structures comprised of vascular endothelial cell cadherins (VECAD) which interact with those of neighbouring cells and assemble a complex containing catenins and actinins to the cytoskeleton [84,85]. When VECs are stimulated by agents that increase cAMP, the activation of PKA and EPAC results in the stabilisation of AJs, decreasing cell permeability [83]. Pharmacological inhibition and siRNA-mediated knockdown identified PDE4D and EPAC1 as the mediators of cAMP-dependent changes in permeability [3]. Interestingly, the use of pharmacological inhibition of PDE4 and knockdown of PDE4D yielded conflicting results. As expected, PDE4 inhibition decreased cell permeability; however, in stark contrast, knockdown of PDE4D increased cell permeability. This was explained by a dual role of PDE4D in this cellular compartment. Not only does the PDE regulate EPAC1 activation by hydrolysing cAMP, but it also acts as the tether, localising EPAC1 to the VECAD structures. The identification of PDE4D’s function as a scaffold identified a novel role for the family of enzymes. Maurice et al. also employed a disruptor peptide to determine the function of the interaction between EPAC1 and PDE4D. Treatment with this peptide reduced VEC permeability to a similar degree as global PDE4 inhibition (Figure 3). The specific disruption of the PDE4D-EPAC1 interaction proved to be a novel method to alter VEC permeability and barrier function.

## 7. Future Directions and Outlook

As described in the present review, the study of PDE4 in the cardiovascular system is centered on the discovery of isoform specific roles within signalosomes. Compartmentalisation of distinct PDE4 isoforms underpin their function and allow low cellular concentrations of the enzyme to have a pivotal role in many areas of cardiac signaling. Future development of new technologies, such as the expansion of novel, highly targeted FRET probes, will allow the effects of PDEs on cAMP signalling to be observed in novel ways that were not previously possible [86]. In conjunction with PDE4 gene therapy, PDE4 activators and novel animal models where inducible dominant negative PD4 constructs are expressed, our understanding of the currently accepted roles of PDEs will be increased, and novel roles at additional microdomains will inevitably be uncovered. This information will undoubtedly drive novel therapeutic endeavors to target this versatile enzyme family for the good of humankind.
